# The Meaning of Hope for Polish Male Patients Dying from Cancer Depending on Their Age: An Interdisciplinary Study with the Use of Osgood’s Semantic Differential Method

**DOI:** 10.3390/jcm13113162

**Published:** 2024-05-28

**Authors:** Bożena Baczewska, Beata Antoszewska, Anna Siwko, Krzysztof Leśniewski

**Affiliations:** 1 Department of Internal Medicine and Internal Medicine in Nursing, Faculty of Health Sciences, Medical University of Lublin, Chodźki 7, 20-093 Lublin, Poland; 2 Department of Special Needs Pedagogy and Resocialization, Faculty of Social Sciences, The University of Warmia and Mazury in Olsztyn, Żołnierska 14, 10-561 Olsztyn, Poland; beata.antoszewska@uwm.edu.pl; 3 Mother Teresa of Calcutta’s Social Welfare Home in Lublin, Głowackiego 26, 20-060 Lublin, Poland; 4 Department of Orthodox Theology, Faculty of Theology, The John Paul II Catholic University of Lublin, Al. Racławickie 14, 20-950 Lublin, Poland

**Keywords:** hope, terminal stage of cancer, semantic differential, palliative and hospice care

## Abstract

**Background/Objectives**: The subject of this article is the reflection on hope—one of the most important predictors and motivators of human actions. Hope is our response to a threat, and it is also the emotion that allows us to overcome hopelessness and to reduce suffering. Hoping is a human capacity with varying cognitive, emotional, and functional dimensions. Psychological, pedagogical (particularly in the framework of special-needs pedagogy and thanatological pedagogy), and theological reflection on hope can be helpful for dying people. The objective of this study was to characterize hope in the semantic space of individuals in the terminal stage of cancer and to verify whether age is a variable that determines this hope. **Methods**: To complete the study, the Osgood semantic differential method was applied, as modified by Polish psychologist Dr. Boguslaw Block (the DSN-3 test). The research technique consisted of a therapeutic conversation. **Results**: Research results show that, in general, those in the terminal stage have positive associations with hope. In all three aspects of the used test, namely the cognitive, emotional, and functional aspects, the highest scores assigned to the perception of hope were obtained from men up to 35 years of age. Depending on the ages of patients, one could observe certain semantic shifts, but they did not prove to be statistically significant. **Conclusions**: Polish males surveyed at the end of life due to cancer generally perceived hope as a supportive force. Therefore, hope can provide emotional support to patients in the terminal stage of cancer and improve their quality of life.

## 1. Introduction

This was the first study, not only in Poland, but also in the world, that explored hope as experienced inside a semantic space measured with the use of the semantic differential. This was confirmed by the perusal of relevant literature carried out by the researchers. Thus far, a pioneering study in Poland on measuring hope with psychometric tools and carried out among patients in the terminal stage of cancer had been completed [1]. That research showed that patients whose condition was referred to as “hopeless” had strong hope, although, medically speaking, they had no chance to recover.

The subject literature [2,3,4] provides a variety of definitions of the concept of hope. In Polish, “hope” is an ambiguous concept and difficult to define precisely. Usually, in existential experiences, especially in traumatic situations, Poles’ hope refers not only to the expectation of some good in the future but also to faith in a personal God and the afterlife. Researchers do not equate hope with optimism, a sense of self-efficacy, or the location of the site of control. Hope basically refers to the future time and has an affective character [5]. Dufault and Martocchio [6] described hope as “a multidimensional force of life” encompassing expectations and opinions that one deems important. Hope, according to Krafft [7], is defined as the sense of having deep trust in the positive consequences of what will happen in the future, especially in difficult circumstances that are outside the individual’s direct control. This author combines two viewpoints, i.e., the cognitive (individualistic) [8] and transcendent/emotional. Krafft focuses on hope connected with faith in someone “greater” than man. In this approach, hope is understood as a phenomenon referring to “auto-transcendent perceptions” and “religious and spiritual experiences”, the “sense of the meaning of life”, and the “experience of closeness in interpersonal relationships”. Paulo Freire underlines that “hope, as an ontological need, demands an anchoring in practice” [9]. Charles Richard Snyder defines hope as “the perceived capability to derive pathways to desired goals, and motivate oneself via agency of thinking to use those pathways” [10]. Snyder’s Hope Theory is helpful to define the word ‘hope’ in relation to both cognitive and affective elements while taking into account abstract and mental goals, different pathways of thinking from the present to the desired future, and freedom of choice. This evaluation tool was intended to measure hope in people.

Without struggle, hope loses its bearings and turns into hopelessness, which can become tragic despair [11]. Hope can also be defined as a subjective projection of the future. Hope was, for the ancient Greeks, considered to be a consolation in states of misfortune, distress, and anguish or when afflicted with some ailment [12].

Terminally ill persons cherish different types of hope, e.g., hope to recover owing to a spontaneous remission of the illness or to supernatural forces, hope for a longer life despite the unfavorable prognosis, or hope to achieve specific goals [13,14,15]. Many patients drawing close to the end of life cling to some kind of hope, e.g., gaining help in dealing with noxious symptoms of the illness and pain, having support and good care, living through another day, finding a sense of life, finding faith and preserving one’s dignity, repairing good relationships with people, having special time with one’s family and closest friends, and hope for a peaceful death and eternal life [16,17,18,19,20].

Schrank, Stanghellini, and Slade [21], in 2008, analyzed a variety of publications dedicated to hope. They were able to identify forty-nine definitions of the concept of hope in the subject literature. The analysis of these various definitions of hope helped them develop their own definition. According to these scholars, hope is a state of man’s consciousness that is a primarily future-orientated expectation of attaining some worthy personal goals, lying in the realm of interpersonal relationships or spirituality. The process of reaching such goals is specific, and it is subjectively evaluated as possible or realistic. Reaching the goal depends on personal activity or characteristics or on external circumstances.

The aforementioned authors distinguished three major components of hope: cognitive (e.g., past memories, evaluation of the chances of achieving some aim, plans concerning the process of reaching the goal), affective (e.g., emotions, feelings), and behavioral (e.g., different types of activity or activities undertaken in connection with the set goal). They also indicated that hope depended on external factors (e.g., availability of resources needed to reach the goal, health care). It is worth noting that in a life-threatening situation, hope can transform into hopelessness. Hope is one of the major predictors and motivators of human action. As indicated by Morse and Doberneck [22], hope is a response to danger, as well as an action giving a chance to overcome hopelessness and to reduce suffering.

Osgood’s semantic differential method can serve as a helpful and valuable tool in examining the meaning of hope for patients in the terminal stage of cancer. This particular tool, differing from other rating scales, can be used to measure associations, attitudes, motivations, or emotions for almost every concept [23]. The fundamental purpose of Osgood’s semantic differential method is to grasp the connotative meaning of concepts by measuring the connotative similarities and differences within and between respondents’ ratings [24,25]. This is especially useful with regard to hope since it provides a sufficient basis for further correlation-based analysis. According to Charles E. Osgood, three recurring attitudes that people use in assessing words and phrases—evaluation (such as “good/bad”), potency (such as “strong/weak”), and activity (such as “passive/active”)—are particularly significant for an analysis of large sets of semantic differential data [26].

### Objective of the Study

The objective of this study was to characterize hope in the semantic space in the three aspects—cognitive, emotional, and functional—within Osgood’s semantic differential method.

The main research goal was to seek an answer to the following question: how do patients in the terminal stage of cancer characterize their hope in the semantic space in the aspects (cognitive, emotional, and functional) indicated by Osgood?

## 2. Materials and Methods

For the purposes of the present study, Osgood’s semantic differential method was used, modified by Polish psychologist Dr. Boguslaw Block, who eventually named it the DSN-3 test. The semantic differential is used to gauge the meaning of concepts. It involves the subject defining a word by selecting semantically opposite adjectives placed at a certain distance from each other. The person being examined then marks a point between the pairs of adjectives, aiming for the one that best represents their own feelings, beliefs, or attitude [27]. The research method used in this study was a therapeutic dialogue [28].

Concerning the DSN-3 tool design, it must be stressed that it was created on the basis of the use of the method of “Competent Judges” (10 psychologists, including 4 doctors of psychology). The judges evaluated the adjectives based on two criteria. The first of these was their common understanding in Polish—meaning that they ensured that the adjectives were part of everyday language and did not exceed the conceptual framework. Secondly, the judges defined their semantic opposition. From the initial list of 87 pairs of opposite adjectives (DSN-1), discriminant analysis selected 40 pairs. Factor analysis was then applied to DSN-2, resulting in the identification of three factors, corresponding to the Osgood nomenclature: (a) truth (cognitive), (b) evaluating (evaluative–emotional), and (c) regulatory (functional). Considering the test’s purpose for individuals in the terminal phase of cancer, it was shortened as much as possible. Consequently, 4 pairs of adjectives were chosen for each factor, totaling 12 pairs with the highest discriminatory power—DSN-3.

Statistical analyses were used to select pairs of opposing adjectives. Following factor analysis, three factors were identified, the first being the cognitive factor, labeled as “truthfulness”. It was found that using only four pairs of opposing adjectives with the highest relevance and a very high “factor input” was sufficient. The severe psychophysical conditions of the individuals for whom this test was intended were considered. Despite the small number of pairs of opposite adjectives, factor analysis was deemed sufficient to describe the concept under study.

The DSN-3 test enables the researcher to describe hope across three dimensions: cognitive, emotional, and functional, as identified by factor analysis. For people in the terminal phase of cancer, 4 pairs of adjectives were selected for each of the dimensions. In the terms of the cognitive aspect of hope, the attitudes of the terminally ill patients to four pairs of objectives (“false-true”, “stupid-wise”, “senseless-sensible”, and “illusory-real”) were taken into account. Four pairs of adjectives were adopted for the emotional aspect of hope (“bad-good”, “gloomy-cheerful”, “hostile-friendly”, and “unhappy-happy”) and for the functional aspect (“debilitating-empowering”, “disturbing-helpful”, “sick-healthy”, “defeated-victorious”). The severe psychophysical condition of the individuals for whom this test was intended was considered. Despite the limited number of pairs of opposite adjectives, factor analysis was deemed adequate for describing the concept under study [29]. A detailed description of the design of this tool is presented in the article titled “The Cognitive Aspect of Hope in the Semantic Space of Male Patients Dying of Cancer”, published in the International Journal of Environmental Research and Public Health by Baczewska et al. [30].

The study was carried out in 17 locations all over Poland. The respondents stayed in stationary hospices (n = 51), home hospices (n = 53), and palliative medicine wards (n = 6). The study group comprised male patients in the terminal phase of cancer whose average age was 60.15 ± 13.53. The average duration of illness for these patients was 35.84 ± 56.96 months. It is worth noting that the largest number of respondents were patients diagnosed with cancers of the sexual system. The respondents, the vast majority, resided in the cities with over 100 s of thousands of inhabitants (n = 41) and in villages (n = 30). A majority lived with family (n = 77) and specified their marital status as married (n = 58) and widowed (n = 27). Concerning the education of the group, it must be pointed out that one-third of these patients had had secondary education (n = 36) and one-fourth had had only vocational education (n = 28). The material status of almost three-fourths of them was satisfactory (n = 51) or good (n = 31). The study was approved by the Bioethics Committee at the Medical University in Lublin (opinion number KE-0254/225/2010). Basic descriptive statistics were applied to describe the collected data, and statistical analysis employed the Kruskal–Wallis test. The research results were processed via the software package Statistica 13.3 (StatSoft Polska Sp. z o.o., Kraków, Poland).

## 3. Results

The results obtained from the study concern both the general perception of hope as well as its three aspects (cognitive, emotional, and functional). In addition, the researchers sought differences in respondents’ overall and aspectual perceptions of hope depending on the age groups to which they belonged. These results are contained in Table 1.

### 3.1. The Respondents’ Hope in the Semantic Space: The Average Global Result

The research results show that, generally, Polish males in the terminal condition have positive associations in relation to hope. The average general result for the whole group of the interviewed men is M = 5.32 ± 1.21, which, according to the research tool, which indicates their rather positive characterization of hope. The results are not strongly positive, which would have indicated that hope was idealized, nor are they rather positive, which would have suggested a risk of depression due to the loss of hope. Particular ratings stretch over the entire scale from 1 to 7. The lowest score (1.0) was interpreted as a decidedly negative perception of hope, being in the semantic space. The highest score (7 points), in turn, formed the evidence to state the existence of definitely positive associations with hope—and the idealization or compensatory re-evaluation of hope.

The results obtained in this survey provided the foundation for indicative statistical norms applicable to the perception of hope among Polish males in the terminal stage of cancer, which were in the range of 4.1 to 6.5. In individual studies, a person’s perception of hope can be assessed against the background of the whole group. The research participants obtained the highest results in the emotional aspect and the lowest in the cognitive aspect.

It is worth noting that not all the men viewed their hope positively. For over 87.27% of all men in the terminal stage of cancer hope was perceived positively or quite positively (4–7). It is noteworthy that 12.73% of the men had rather pejorative associations with hope (scores between 1 and 3.99). Indifferent or ambivalent associations with hope were declared by 28.18% of all the men participating in the study (range: 3–4.99).

The average general score of associations with hope in the semantic space with respect to the cognitive aspect assigned by Polish males dying of cancer was 5.13 ± 1.42. According to the research tool, this result attests to the positive perception of hope. The results enable us to determine a statistical norm of the connotative concept of hope among men in the terminal stage of cancer. The scores in the range of 3.71–6.55 indicate the typical location of hope in the cognitive aspect within the semantic space.

### 3.2. The Cognitive Aspect of Hope in the Opinion of the Respondents

The perception of hope in the cognitive aspect by the entire sample of Polish male patients was scored mainly at 6–7 points (n = 38, 34.55%) and at 5.0–5.99 (n = 36, 32.73%), which correspond to a definitely positive and positive view of hope, respectively. Nearly every sixth research participant (16.36%) perceived hope quite positively. The overwhelming majority of the men, that is, 92 (83.6%), showed a positive attitude to hope in the cognitive aspect, assigning to it such adjectives as “true”, “wise”, “sensible”, and “real”. In contrast, 18 (16.36%) of the surveyed men defined hope using pejorative adjectives like “false”, “stupid”, “senseless”, and “illusory”. This is reflected by the results within the class categories from 1.0 to 3.99. Three of the participants associated hope seen from the cognitive perspective with extremely negative associations (n = 3, 2.73%) and chose such adjectives as “definitely stupid”, “false”, “senseless”, and “illusory”. This is probably a consequence of their recent disillusionment—painful disappointment because of failing to achieve the desired goals and the resulting frustration.

### 3.3. The Emotional Aspect of Hope in the Opinion of the Respondents

The perception of hope in the emotional aspect by the male patients was scored M = 5.50 ± 1.25, which, according to the applied research tool, is positive, although if “common sense is applied”, they are “in a hopeless situation”. The scores given by the overwhelming majority are in the range of 4.25–6.75. Thus, they are in the range of a statistical norm for this group of men in the terminal stage of cancer.

The perception of hope in the emotional aspect among the surveyed men was most often scored in the range of 6–7 (45.45%). This result proves a definitely positive attitude to hope in this aspect. On average, one in four interviewees (26.36%) had a rather positive attitude to hope, and one in five (21.82%) had a positive one. The least frequent attitudes were negative, in the range of 2.0–2.99 (1.82%), and very negative, lying between 1.0 and 1.99 (1.82%). When selecting descriptive words to refer to hope in the emotional aspect, the men more often chose positive adjectives. For 93.6% of the men, hope brought associations with warmth and something good. They perceived hope as “good”, “cheerful”, “friendly”, and “happy”. However, 6.4% of the men admitted that for them, hope had a negative emotional connotation and appeared as “bad”, “gloomy”, “hostile”, and “unhappy”. This could be a consequence of lost hopes, probably connected with poor interpersonal relationships.

### 3.4. The Functional Aspect of Hope in the Opinion of the Respondents

In a person who is at the end of their life, hope in the functional aspect manifests itself in the way he/she struggles with this difficult situation. The choice of the affirmation or negation level of adjectives describing the functional aspect of hope indicates whether and how the respondent copes with suffering and the fear of death. This study’s results show that in the functional aspect of the semantic space, hope becomes a supporting force. The arithmetic mean was 5.33 ± 1.32 SD, which leads to the conclusion that hope is perceived as being neither a weak force nor an omnipotent or magical power that could guarantee miraculous recovery, although even such a perception of hope could be detected among the dying men in our survey (a score given by a research participant surpasses 6.7). If the score was below 4.0, this meant that the respondent did not feel the supporting force of hope. The perception of hope in the functional aspect among the surveyed men was most often given scores between 6.0 and 7.0 (39.09%). This means that these participants treated hope as a definitely supportive force while 25.45% saw it as a supportive force in their struggle with the problems, condition, symptoms, etc. encountered due to the terminal stage of cancer. Moreover, 53.63% of the interviewed men had positive and quite positive associations with hope in this aspect, which substantiates the conclusion that hope was a supportive force helping them to live through this difficult time. This is mirrored by the perception of hope that supports and helps one overcome the stress perpetrated by the terminal stage of neoplastic disease.

It was interesting to observe that 7.28% of the analyzed men had a pejorative attitude to hope seen in the functional aspect. Four of the respondents (3.64%) saw hope in a distinctly negative manner, suggesting that it weakened them and made it more difficult for them to recover, which meant that this kind of hope was insane and defeatist. This location of hope in the semantic space reveals the depression and despair held by these terminally ill men and, in a certain sense, proves that they had given up on future life and that they realized they did not hold the power to change the outcome of their illness. This was also a manifestation of being unadjusted to having such an illness. In this condition, a patient typically experiences the overwhelming sense of being personally powerless and hopeless, which means that one needs help from others, especially from carers and close family. Noteworthy is the fact that the vast majority of the respondents (93%) perceived hope as a strength supporting them in this extremely difficult psycho-physical state and existential and spiritual condition.

### 3.5. Differences in the Perception of Hope in the Semantic Space Due to the Age of the Respondents

The men included in the study were divided into four main age groups, taking into account their personal life and professional activity throughout their life spans, i.e., from 18 to 35—youth and early professional career, 36 to 50 years—early maturity and peak professional activity, 51 to 65 years—late maturity and a period of late professional activity, and over 65 years of age—retirement. Because there were only six men in the first age bracket, no more than 35 years old, the results need to be treated as indicative.

#### 3.5.1. Hope of Men in Young Age and Early Professional Career (18–35 Years Old)

In the youngest age group, the results were clearly shifted towards the positive part of the scale. The average result of the perception of hope by men aged 18–35 years was 5.83 ± 0.80, which means that the men in this age class had demonstrably positive associations with hope. The lowest degree of affirmation, although still positive (5.64) in the cognitive aspect, was demonstrated by the men as regards hope in the true and false perspective (3–5.75). In the emotional aspect (in the scope of concretization: “good-bad”), they evaluated hope as definitely good, which was rooted in the conviction that hope was a power helping one live and deal with suffering, as well as a means to overcome the stress caused by the awareness of death approaching rapidly and inevitably at such a young age. The standard deviation implicates the quite high homogeneity of the group and the way they experienced hope.

#### 3.5.2. Hope of Men in the Stage of Early Maturity and Highest Professional Activity (36–50 Years Old)

For this age group, too, the results shifted towards high scores. However, the inter-group variation was greater. The average general result for the whole group was 5.26 ± 1.13, which means that most results were within the range of 4.13–6.39. This means that the association with hope shared by these men was positive. As with the group of men aged 18–35 years, these men, between 36 and 55 years of age, maintained that hope had the least positive associations in the cognitive context (5.03) and the highest in the emotional aspect (5.70).

#### 3.5.3. Hope of Men in the Late Maturity Period and Late Professional Career (51–65 Years)

The results were also shifted towards the positive part of the scale in this group of men. However, the scattering of results was greater, which implicates large differences between individual respondents in this age group (M = 4.09–6.63). The average general result of hope seen in the semantic space of the men surveyed was 5.36 ± 1.27, which means that a positive perception of hope was held. Single results, however, oscillated from definitely negative to definitely positive in the semantic space. This means that some of the respondents considered hope definitely positive, while others considered it definitely negative. The surveyed group of men aged 51 to 65 years achieved the highest average results in the emotional aspect (5.55) and functional aspect (5.42), which indicates the positive perception of hope in these aspects, while the lowest, but also positive, average scores were assigned to hope in the cognitive aspect (5.12).

#### 3.5.4. Hope of Men in the Retirement Age (Over 65 Years Old)

In this group of men, the results were also shifted towards the positive part of the scale. However, the median was lower than the arithmetic mean, which indicates that the results were not inclined towards high scores as in the previous age groups. Furthermore, the scattering of the results was slightly smaller than among the men still active professionally (M = 3.99–6.43). The average general result concerning the perception of hope among the men aged over 65 years was 5.21 ± 1.22. The most varied results appeared in the cognitive aspect, in the space from “false” to “totally genuine hope”. The least diverse results emerged in the functional aspect, where hope was seen as a supportive healthy force helping one to deal with illness and current existential problems.

### 3.6. Examples of Extreme Results

**Figure 1:**
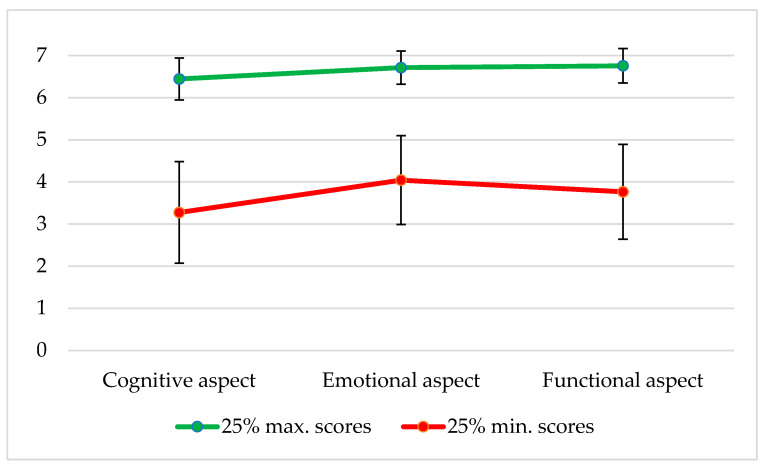
Differences in the hope profiles of the male patients resulting from their extreme scores on the DSN-3 test.

The research results revealed very extreme attitudes or ways of experiencing and cherishing hope. The semantic differential method applied in this study enables capturing the projection of one’s mental states and feelings for the analyzed reality. This explains why it is worth paying attention to extreme cases. They are presented below, in Figure 1.

The profile of hope among men with the highest scores on the DSN-3 survey was characterized by very high results in all the aspects: cognitive, emotional, and functional. The profile suggests that the interviewed men in the terminal stage of cancer had the image of hope as “true”, “wise”, “reasonable”, “real”, “good”, “cheerful”, “friendly” and “happy”, “supportive”, “helpful”, “healthy”, and “victorious”. This profile reflects the tendency of re-evaluating hope in the direction towards its idealization. This may indicate both an accepting expectation of what is inevitable—peaceful and dignified dying—and death itself. This may also result from profound religious faith, the expectation of an encounter with a loving God who endows His sons and daughters with eternal happiness. Another reason that should be considered is that hope experienced in an extremely positive manner can mask a hidden fear protecting one from suffering and death, which are shunned behind compensatory, idealized, childlike hope.

In turn, the profile of hope demonstrated by men scoring the lowest on the DSN-3 test was characterized by the lowest average scores on the scale in the emotional aspect and scores below the theoretical mean (Mt = 4.0) in both cognitive and functional aspects. The average result should be interpreted as an indifferent or ambivalent attitude to hope. The study showed that hope was perceived the most pejoratively in the cognitive aspect. This may indicate the patients’ attitude to hope as the proverbial “mother of fools”. In this sense, hope had not fulfilled the expectations of the surveyed men. It was a deceptive, disturbing, and debilitating force. It led to undertaking various, often radical, struggles with cancer in its terminal stage, contrary to a common-sense assessment of one’s existential situation and therapeutic options. The research results can be interpreted as manifestations of depression of different severity levels; surrender to a state of desperate hopelessness, powerlessness, and complete loss of the purpose of life; and the acceptance of inevitable death.

**Figure 2:**
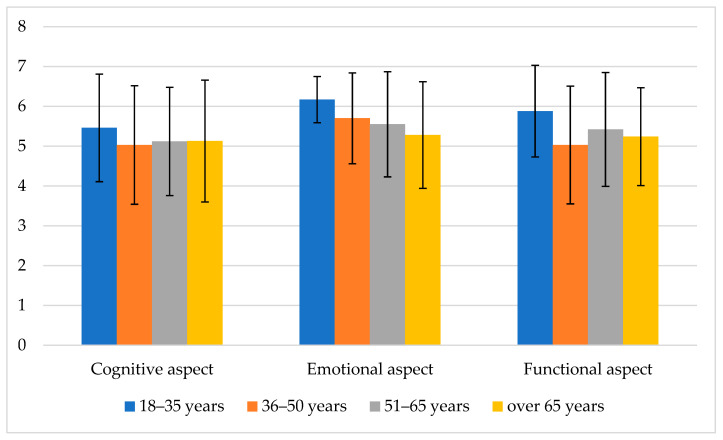
Profiles of hope of Polish male patients in different age groups as measured by way of the DNS-3 test.

In all the three aspects (cognitive, emotional, and functional) of the perception of hope in the distinguished age groups of Polish males dying of cancer, it was possible to observe semantic shifting. Still, while, due the different ages and life activity levels of the patients, one might expect significant differences in how these men experienced hope, these differences were not significant, as seen in Figure 2.

In all the three aspects, the highest scores with respect to the perception of hope were given by the men up to the age of 35 years, while those between 36 and 50 years, between 51 and 65 years, and over 65 years gave slightly lower scores. However, it is uncertain whether these differences were significant enough to signify a notable regularity for this very specific group of male patients in the terminal stage of neoplastic disease. To clarify this, a statistical analysis of the differences was conducted. None of the aspects of the connotative meaning of hope in the four age groups with a different number of non-parametric variables as measured with the Kruskal–Wallis test showed statistical significance (see Table 2).

It can therefore be concluded that the different shades of the meaning of hope declared by Polish males in the terminal stage of cancer have a similar importance or location in the semantic space. Thus, it is impossible to determine regularity in how the connotative meaning of hope changes depending on the ages of men dying of cancer. It is only justifiable to claim that the meaning and role of hope among Polish males terminally ill due to cancer do not depend directly on their ages but can be affected by other factors as well.

## 4. Discussion

The results of the study allow us to conclude that the respondents positively perceive their hope in all its aspects at a high level. The respondents perceive hope as a supportive force enabling them to cope with all the problems arising from painfully being at the last stage of life, which is always accompanied by anxiety, fear, and panic regarding the unknown [31,32,33]. Therefore, it can be considered that their hope is mature in a special way, which can be seen in the cognitive, emotional, and functional spheres. If the vast majority of respondents perceive hope in the cognitive sphere as “true”, “wise”, and “sensible and real”, and in the emotional sphere as “good”, “cheerful”, “friendly”, and “happy”, then the logical consequence is that hope in the functional aspect is, for them, an empowering and helpful force and, at the same time, a healthier and victorious power that makes them not fall into desperation, apathy, and despair. This makes it easier for them to take action aimed at solving various problems caused by their fatal disease. Based on the research conducted, it seems to be a reasonable view that even in the last stage of cancer, hope can play a significant role. While the medical prognosis may indicate limited biological viability or exhausted treatment options, hope can provide a sense of purpose, meaning, and resilience [18,34,35]. Hence, “wise”, “true”, “real”, and “meaningful” hope can help patients and their loved ones cope with the complex emotional and existential challenges that come with the terminal stage of cancer [36]. Death is then treated as the completion and culmination of Earthly life. In palliative care, this kind of death is described as “good” and “beautiful”. If the dying person is experiencing “good despondency”, then, although it is not a joy and something desirable for him to approach the inevitable end of Earthly life, he reconciles himself to this fact and cooperates with all those who take care of him.

The results presented in this article are part of comprehensive studies concerning the multifaceted assessment of hope among terminally ill cancer patients undergoing palliative and hospice care in Poland and are a continuation of the studies published by Baczewska et al. [1,37,38,39,40]. The aim of this study has been to characterize hope in the semantic space of men in the terminal stage of cancer, taking into account their ages [41,42]. More detailed specification on hope as experienced by patients dying of cancer and the determination of the factors that can influence how hope is perceived can be helpful in diagnosing patients at higher risk of losing hope and more prone to falling into the feelings of hopelessness and powerlessness that are symptomatic of being in a state of despair [43]. Having precisely indicated the factors related to the degree of affirmation of hope in the semantic space, it will be possible to plan holistic interdisciplinary care for patients in the terminal stage of neoplastic disease [44].

Hope in the semantic space of the whole group of male patients included in the study (who are in the terminal stage of cancer) was generally perceived positively. The respondents had the most positive perception of hope in the emotional aspect while having the least positive outlook on hope in the cognitive aspect. The analysis of the profile of hope regarding the cognitive aspect demonstrates that hope for the respondents is more “true” than “false”, more “wise” than “stupid”, more “sensible” than “senseless”, and more “real” than “illusory”. In the emotional aspect, the surveyed men declared that hope was more “good” than “bad”, more “cheerful” than “gloomy”, more “friendly” than “hostile”, and more “happy” than “unhappy”. In the functional aspect, hope was perceived as more “empowering” than “debilitating”, more “helpful” than “disturbing”, more “healthy” than “sick”, and more “victorious” than “defeated”.

The attitudes to hope among the surveyed men in every age group were similar and testified to positive associations. The highest degree of affirmation among any of the age groups could be noticed in terms of the emotional aspect. The study did not show any significant differences in the general perception of hope in the semantic space depending on the ages of the respondents. It can therefore be concluded that age does not play a significant role in the perception of hope in the semantic space of the men included in the survey. “Wise”, “true”, “real”, and “meaningful” hope can provide patients with the strength and resilience to face their situation while “false”, “deceptive”, and “meaningless” hope can exacerbate suffering and despair. It is important for healthcare providers and family and friends to support patients in cultivating a sense of wise hope, which acknowledges the reality of their situation while also fostering a positive outlook and a sense of purpose. This can help patients make the most of their remaining time and find meaning and fulfillment in their lives even in the face of terminal illness. Conversely, “false” hope can lead to a sense of denial and avoidance, preventing patients from fully engaging with their situation and seeking appropriate care and support. This can ultimately worsen their physical and emotional well-being and diminish their quality of life. By recognizing the difference between “wise hope” and “false hope”, healthcare providers and loved ones can better support patients in navigating their journey with terminal illness and promoting their well-being and quality of life.

The study described in this article was a pioneering research effort. Studies conducted to date have mostly dealt with the basic notion of hope as held by patients with neoplastic disease [44]. For example, investigations completed in Germany and in Switzerland indicate that the level of basic hope of patients with cancer is high. This is true even with regard to those patients who are at an advanced stage of cancer. The mentioned studies, performed with the use of the Perceived Hope Scale, showed a significant positive correlation between overall levels of hope and the ways in which people cope with traumatic situations [45].

It would be very helpful to develop an interdisciplinary bio-psycho-social–spiritual care strategy to learn how hope is understood and characterized in all aspects of thought of patients with terminal cancer [46]. By understanding and analyzing the causes that decrease stress levels and increase the level of positively directed hope, healthcare providers can plan integral care that alleviates symptoms characteristic of the terminal stage of cancer and better prepare the patient approaching the end of their life [47].

The analysis of relevant literature has shown that there have been no studies addressing the specific issue being discussed. This indicates a gap in the existing research. Therefore, it is recommended to conduct more comprehensive research on the topic. This would involve a more in-depth investigation into the issue at hand. The research gap pertains to the perception of hope in the semantic space by patients in the terminal stage of cancer. This suggests that the focus of the research should be on the concept of hope and how it is perceived by these patients. The research would explore how the perception of hope affects the life attitudes, emotional functioning, and social functioning of patients in the terminal stage of cancer. Such research would have implications for understanding the psychological and social aspects of dealing with terminal illness. It would be supportive to examine how the perception of hope influences the quality of relationships that patients have with themselves and others and how it affects their ability to cope with the stress of suffering and imminent death.

Considering the needs of patients in the terminal stage of cancer, it seems rational to conduct further studies on both basic hope and hope in the semantic space. Owing to an analysis of the cognitive, emotional, and functional aspects of hope in the semantic space, it should be possible to gain better insight into the patient’s condition (bio-psycho-social–spiritual) and the resulting problems associated with the process of dying; this will also enable caregivers to provide more effective care [48,49].

### Limitations of the Study

Nevertheless, it is important to consider that carrying out this kind of research necessitates the precise selection of patient inclusion and exclusion criteria. Among the inclusion criteria for the study should be an age above 18, the patient’s ability for logical communication, their informed consent, and being in the terminal stage of cancer. It should also be mentioned that some patients refused to participate in the study because they felt too weak, and some patients did not complete the study due to a sudden deterioration of their health.

## 5. Conclusions

Based on the study, the following conclusions were drawn:

- The men surveyed at the end of life due to cancer generally saw hope as a source of support. The average score indicates that their hope was not weak; nor was it seen as a magical guarantor of recovery. Instead, it was viewed as preparation for a dignified death.
- For the majority of respondents, the cognitive aspect of hope involved expecting to achieve their goals in the future, along with accepting the inevitability of suffering and death, and recognizing that a good and dignified death would occur at the appropriate time.
- For the majority of respondents nearing the end of life, hope in the emotional aspect was not illusory or based on magical thinking that would hinder their adaptation to reality. Instead, it served a constructive purpose, offering a sense of security and psycho-social–spiritual comfort to help them come to terms with death.
- Hope, in its functional aspect, was an empowering, supportive, healthy, and triumphant force for most respondents, enabling them to direct their efforts more effectively toward resolving the ongoing problems resulting from the terminal phase of cancer. This perception of hope facilitated their relationships with loved ones, enhanced their experience of being loved, and provided them with a sense of life’s uniqueness, along with a serene acceptance of the unavoidable approach of the end of earthly existence.

## 6. The Importance of Hope for Patients in the Terminal Stage of Cancer in Practical Terms

- Hope has the potential to offer emotional support to patients and their families, assisting them in dealing with the distress and uncertainty that come with a terminal diagnosis.
- Hope can enhance the quality of life by fostering a positive outlook and motivating patients to participate in various activities.
- Hope can assist patients in discovering meaning and purpose in their lives, even when confronted with illness and death. This is particularly significant for individuals who are wrestling with existential questions regarding the meaning of life and death.
- Hope can cultivate resilience, empowering patients to confront the challenges of their illness with courage and determination.
- Hope can impact decision making by motivating patients to consider all available options for care and treatment, including palliative and hospice care, and to make choices that are in line with their values and preferences.

It is important to note that while hope can be a valuable resource, it should be balanced with realistic expectations and an understanding of the limitations of medical treatment. This is where the concept of “healthy hope” comes to play. “Healthy hope” involves acknowledging the reality of the situation while also maintaining a sense of optimism and possibility.

## Figures and Tables

**Table 1:** Perceptions of hope in general and in cognitive, emotional, and functional aspects by male patients in the terminal stage of cancer depending on age—descriptive statistics.

Age	Descriptive Statistics	Aspects of Hope According DSN-3			
		Cognitive Aspect	Emotional Aspect	Functional Aspect	Global Results
All patients (N = 110)	M	5.13	5.50	5.33	5.32
	SD	1.42	1.28	1.35	1.21
	Me	5.50	5.50	5.50	5.46
	Min	1.0	1.0	1.0	1.0
	Max	7.0	7.0	7.0	7.0
18–35 years (N = 6)	M	5.46	6.17	5.88	5.83
	SD	1.35	0.58	1.15	0.80
	Me	5.88	6.38	6.25	6.13
	Min	3.0	5.0	4.0	4.33
	Max	6.75	6.50	7.0	6.50
36–50 years (N = 15)	M	5.03	5.70	5.03	5.26
	SD	1.49	1.14	1.48	1.13
	Me	4.75	5.50	5.0	5.42
	Min	2.25	4.0	1.50	3.75
	Max	7.0	7.0	7.0	7.0
51–65 years (N = 49)	M	5.12	5.55	5.42	5.36
	SD	1.36	1.32	1.43	1.27
	Me	5.50	5.75	5.50	5.75
	Min	1.0	1.0	1.0	1.0
	Max	7.0	7.0	7.0	7.0
over 65 years (N = 40)	M	5.13	5.28	5.24	5.21
	SD	1.53	1.34	1.23	1.22
	Me	5.50	5.13	5.0	5.17
	Min	1.0	1.75	1.50	2.33
	Max	7.0	7.0	7.0	7.0

**Table 2:** Significance of differences in the connotative meaning of hope depending on the age of the surveyed men as measured with the DSN-3 test.

Aspects of Hope According DSN-3	Age Groups (Median Results)				Kruskal–Wallis Test	
	18–35	36–50	51–65	Over 65	H	*p*
Cognitive aspect	5.88	4.75	5.50	5.50	0.558	0.906
Emotional aspect	6.38	5.50	5.75	5.13	2.726	0.436
Functional aspect	6.25	5.00	5.50	5.00	2.597	0.458
Global result M_GL_	6.13	5.42	5.75	5.17	1.700	0.637

## Data Availability

The data presented in this study are available on request from the corresponding author.
